# Gefitinib enhances sensitivity of endometrial cancer cells to progestin therapy via dual-specificity phosphatase 1

**DOI:** 10.18632/oncotarget.23264

**Published:** 2017-12-15

**Authors:** Yuan Yang, Jingyi Zhou, Xiaoping Li, Lijun Zhao, Yuan Cheng, Yanying Lin, Jiaqi Wang, Lihui Wei, Yafeng Dong, Jianliu Wang

**Affiliations:** ^1^ Department of Obstetrics and Gynecology, Peking University People’s Hospital, Beijing, China; ^2^ The First Hospital of Lanzhou University, Lanzhou, China; ^3^ Obstetrics and Gynecology Department, University of Kansas School of Medicine, Kansas City, KS, USA

**Keywords:** endometrial cancer, gefitinib, MPA, DUSP1, targeted therapy

## Abstract

In this study, we investigated if Gefitinib, an epidermal growth factor receptor (EGFR) inhibitor, augments endometrial cancer (EC) therapy with medroxyprogesterone acetate (MPA). Combined treatment with Gefitinib plus MPA decreased the proliferation and invasiveness of the Ishikawa and RL952 EC cell lines more effectively than MPA treatment alone. Moreover, combined treatment with Gefitinib plus MPA reduced growth of EC xenografts in Balb/c nude mice more than either Gefitinib or MPA alone. The therapeutic efficacy of combined Gefitinib plus MPA treatment was dependent on expression of dual-specificity phosphatase 1 (DUSP1). DUSP1 knockdown in Ishikawa cells treated with Gefitinib plus MPA showed greater proliferation and invasiveness than parental Ishikawa cells treated similarly. EC cells treated with the combination of Gefitinib plus MPA also showed DUSP1-dependent reductions in phospho-ERK1/2 and increases in E-Cadherin. Thus, Gefitinib appears to DUSP1-dependently enhance the therapeutic efficacy of progestin in EC cells.

## INTRODUCTION

Endometrial cancer (EC) is the most frequently diagnosed gynecological malignancy worldwide, with more than 100,000 newly diagnosed EC cases reported in China and United States annually [1–3]. About 3-5% of women diagnosed with EC are < 40 years of age, and 70% of these women are nulliparous [4]. The 5-year survival rate of early-stage EC patients is 91% [5], but, it decreases to 20-50% in EC patients with advanced and metastatic disease [6]. The current first-line therapy for advanced and metastatic EC patients is a combination of carboplatin and paclitaxel, which has a response rate of about 40-50% in chemotherapy-naive patients and is accompanied with severe adverse effects [7–10]. Therefore, there is an urgent need for novel therapeutic options for nulliparous and advanced EC patients that are effective and without adverse effects.

Progestins such as medroxyprogesterone acetate (MPA) and megestrol acetate (MA) have been used for hormonal therapy in EC since 1970s to induce differentiation in normal glandular endometrial epithelium [11]. Advanced EC patients treated with tamoxifen and alternating weekly cycles of MPA show a median progression-free survival (PFS) of 3 months and median overall survival (OS) of 13 months [12]. These data demonstrate the potential of combined tamoxifen plus MPA therapy for patients with advanced or recurrent EC, regardless of tumor grade or hormone receptor status. Progestins are also used in hormonal treatment for young EC patients to preserve fertility [13]. However, progestin therapy is limited because 30% of EC patients exhibit resistance to hormonal therapy [14]. Furthermore, overexpression of epidermal growth factor receptor (EGFR) is associated with progestin resistance and poor prognosis of advanced stage EC patients [15]. Gefitinib is a potent, specific inhibitor of the EGFR pathway that binds to the ATP binding site in the EGFR tyrosine kinase domain with a greater affinity than ATP, thereby suppressing EC progression [16].

In our previous study, we showed that dual-specificity phosphatase 1 (DUSP1) expression was a negative prognostic indicator in EC [17]. Moreover, DUSP1 deficiency induces EC progression by activating the MAPK/ERK signaling pathway [18]. In EC patients receiving progestin therapy, DUSP1 overexpression correlates with better prognosis [17, 18]. High DUSP1 expression is also inversely correlated with JNK activity in breast cancer [19]. In hepatocellular carcinoma, high DUSP1 expression correlates with better prognosis [20]. In pancreatic ductal adenocarcinoma, DUSP1 enhances anti-tumor effects of Gemcitabine by promoting apoptosis and suppressing tumor growth and angiogenesis [21].

Many studies have shown that MPA is a key inhibitor of EC progression [11]. MPA treatment induces DUSP1 overexpression, which decreases tumor progression in EC [18]. However, the effects of Gefitinib on DUSP1 expression in MPA-based EC therapy have not been studied. Therefore, we investigated if Gefitinib augmented MPA-based EC therapy via DUSP1.

## RESULTS

### Gefitinib augments anti-tumor effects of MPA in EC cells

**Figure 1:**
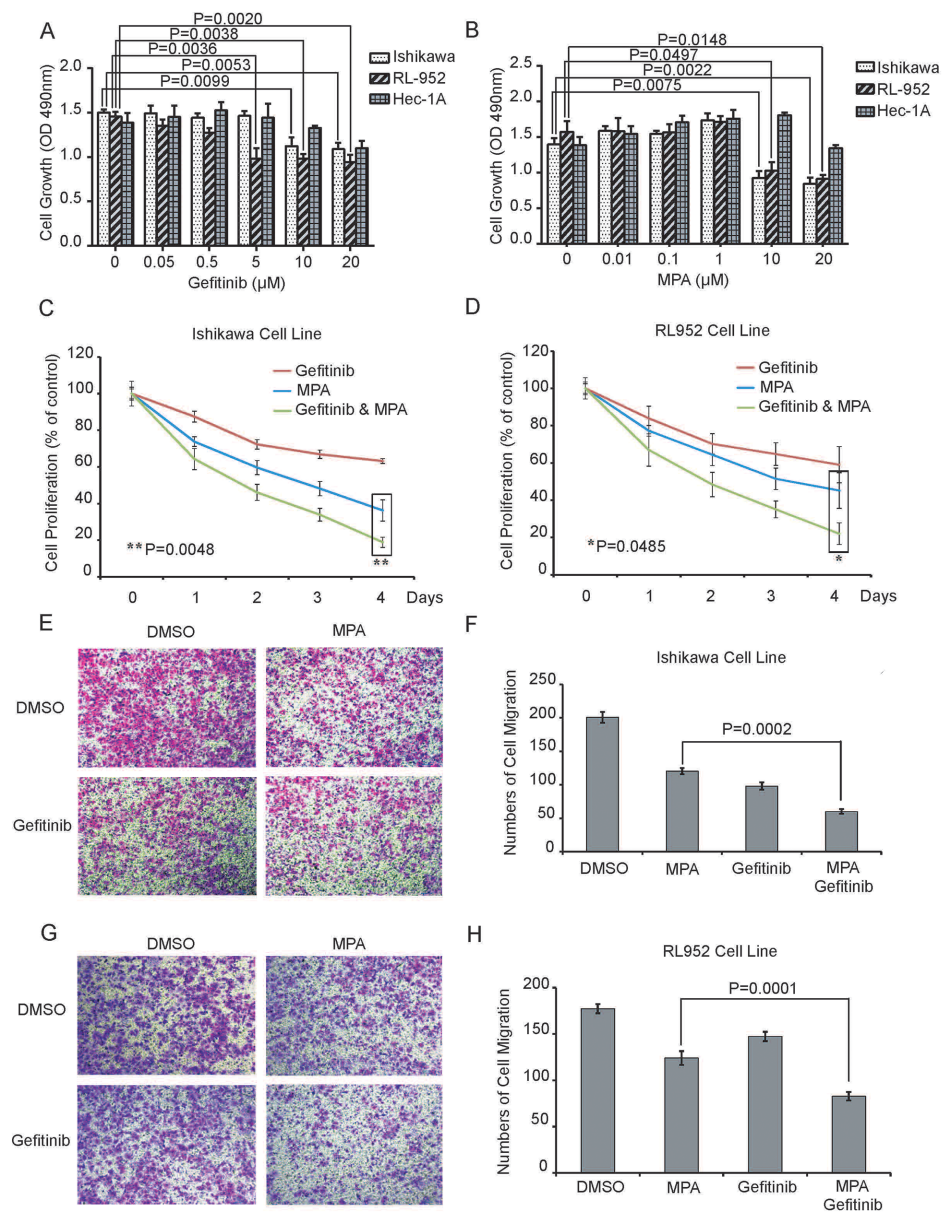
Combined Gefitinib and MPA treatment decreases proliferation and migration of EC cell lines **(A-B)** CCK-8 assay shows proliferation of Ishikawa, RL952 and Hec1A endometrial cancer cells treated with different doses (0, 0.05, 0.5, 5, 10 and 20μM Gefitinib; 0, 0.01, 0.1, 1, 10 and 20μM MPA) of Gefitinib or MPA for 24 h. As shown, treatment with Gefitinib and MPA decreases proliferation of EC cells in a dose-dependent manner. **(C-D)** Growth curve of Ishikawa and RL952 cells treated with 5μM Gefitinib, 10 μM Gefitinib, 10μM MPA or 10μM MPA plus 5 μM or 10μM Gefitinib for four days. CCK-8 assay was performed on all the 4 days at 490 nm. Y-axis indicates the percentage of cell growth relative to DMSO-treated EC cells. **(E-F)** Histogram plots show Transwell migration rates of Ishikawa cells treated with 10μM Gefitinib, 10μM MPA or 10μM MPA plus 10μM Gefitinib for 24h. **(G-H)** Histogram plots show Transwell migration rates of Ishikawa cells treated with 5 μM Gefitinib, 10μM MPA or 10μM MPA plus 5 μM Gefitinib for 24h. Note: In 1E-H, graph represents average number of cells determined in five high power fields (x 200) for each condition in duplicate. Error bars represent standard deviation from triplicate experiments.

We performed CCK-8 assay to evaluate proliferation of Ishikawa, RL952 and Hec1A cell lines that were treated with different doses (0, 0.05, 0.5, 5, 10 and 20μM Gefitinib; 0, 0.01, 0.1, 1, 10 and 20μM MPA) of Gefitinib and MPA for 24 h. We observed a dose-dependent decrease in proliferation of Ishikawa and RL952 cells treated with Gefitinib and MPA, whereas Hec1A cell proliferation was inhibited by very high dose (20μM) of Gefitinib or MPA (Figure 1A-1B). Furthermore, Ishikawa and RL952 cell growth was inhibited by 5μM and 10 μM Gefitinib, whereas 10 μM MPA decreased growth of Ishikawa and RL952 cells (Figure 1A-1B). Therefore, for further experiments, we selected 5μM and 10 μM Gefitinib as well as 10 μM MPA doses to treat Ishikawa and RL952 cells. Hec1A cell line was excluded from further experiments because it was resistant to low doses of the two drugs.

Since EGFR signaling could involve in progestin-resistance of EC [22], we evaluated growth of Ishikawa and RL952 cells treated with 5μM Gefitinib plus 10 μM MPA or 10 μM Gefitinib plus 10 μM MPA for four days. Combined treatment of Gefitinib and MPA was more effective in inhibiting growth of EC cells than Gefitinib or MPA treatments alone (Figure 1C-1D).

Next, we examined the effect of combined Gefitinib and MPA treatment on invasiveness of EC cells by performing Transwell migration assay. We observed that combined treatment of 10 μM MPA with 5μM or 10μM Gefitinib decreased migration of Ishikawa and RL952 cell lines than MPA or Gefitinib treatments alone (Figure 1E-1H). Together, these results demonstrate that Gefitinib enhances the suppression of growth and migration of EC cells of MPA.

### Gefitinib promotes MPA-mediated inhibition of EC xenograft tumors in nude mice

**Figure 2:**
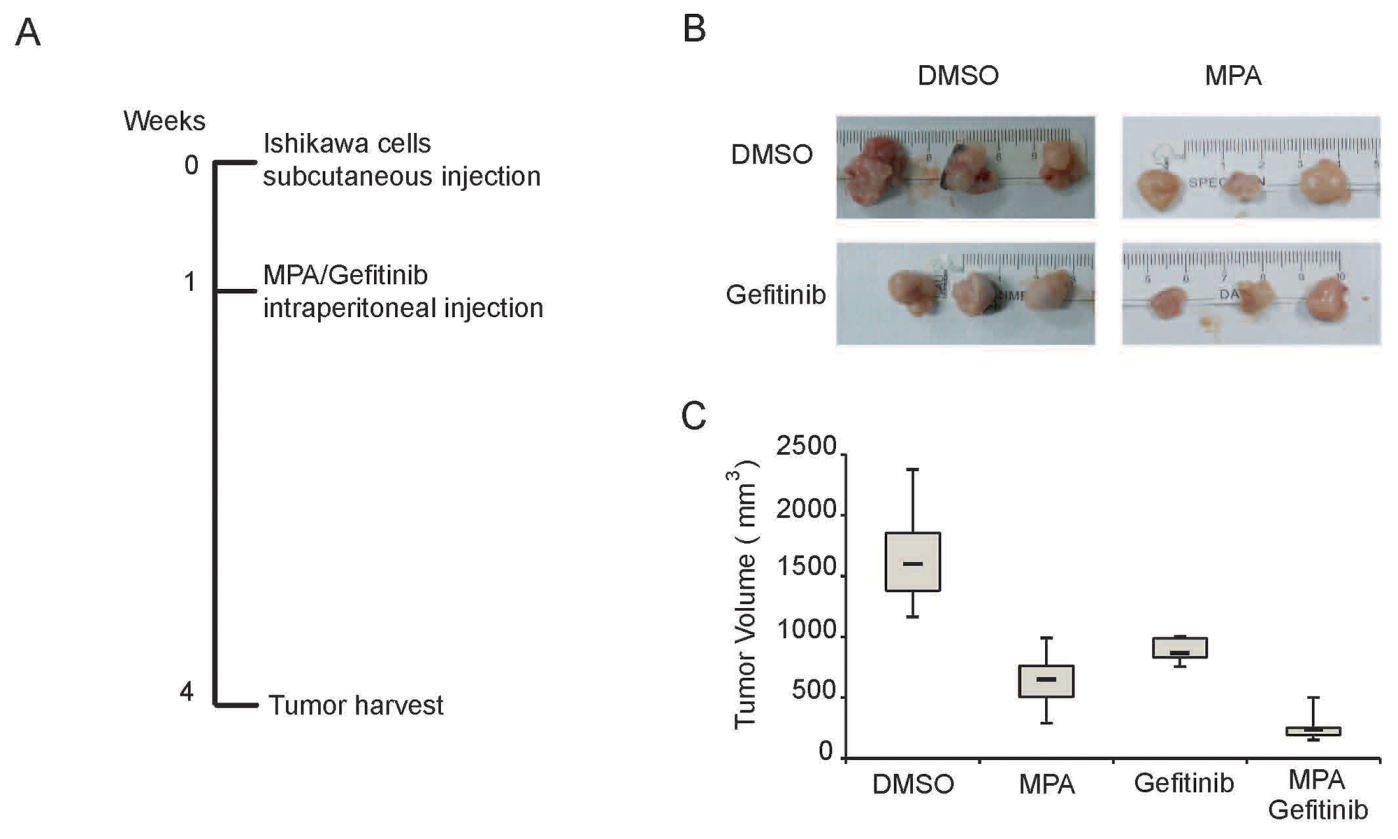
Gefitinib enhances MPA-mediated tumor suppression in the EC xenograft model in nude mice **(A)** Representative images of tumors derived from Ishikawa cells subcutaneously injected into Balb/c nude mice at 3 weeks after treatment with (i) 100μl DMSO; (ii) 500 mg/kg MPA; (iii) 250 mg/kg Gefitinib; or (iv) 500 mg/kg MPA plus 250 mg/kg Gefitinib. **(B)** Representative weight and volume of xenograft tumors derived from Ishikawa cells in Balb/c nude mice treated with (i) 100μl DMSO; (ii) 500 mg/kg MPA; (iii) 250 mg/kg Gefitinib; or (iv) 500 mg/kg MPA plus 250 mg/kg Gefitinib. **(C)** Histogram plots show xenograft tumor volumes in Balb/c nude mice treated with (i) 100μl DMSO; (ii) 500 mg/kg MPA; (iii) 250 mg/kg Gefitinib; or (iv) 500 mg/kg MPA plus 250 mg/kg Gefitinib.

Next, we examined if Gefitinib enhances the anti-tumor activity of MPA *in vivo* by subcutaneously xenografting Ishikawa cells into nude mice and treating them with intraperitoneal injections of DMSO or MPA with or without Gefitinib for 3 weeks. After 3 weeks, we observed that combined treatment of MPA and Gefitinib decreased tumor volume more effectively than MPA or Gefitinib treatments alone (Figure 2A-2C). Moreover, there was no significant loss of body weight or other symptoms of toxicity in nude mice treated with MPA and Gefitinib. These results demonstrate that Gefitinib enhances anti-tumor effects of MPA *in vivo*.

### DUSP1 knockdown blocks therapeutic effects of combined Gefitinib and MPA treatment in EC cells

**Figure 3:**
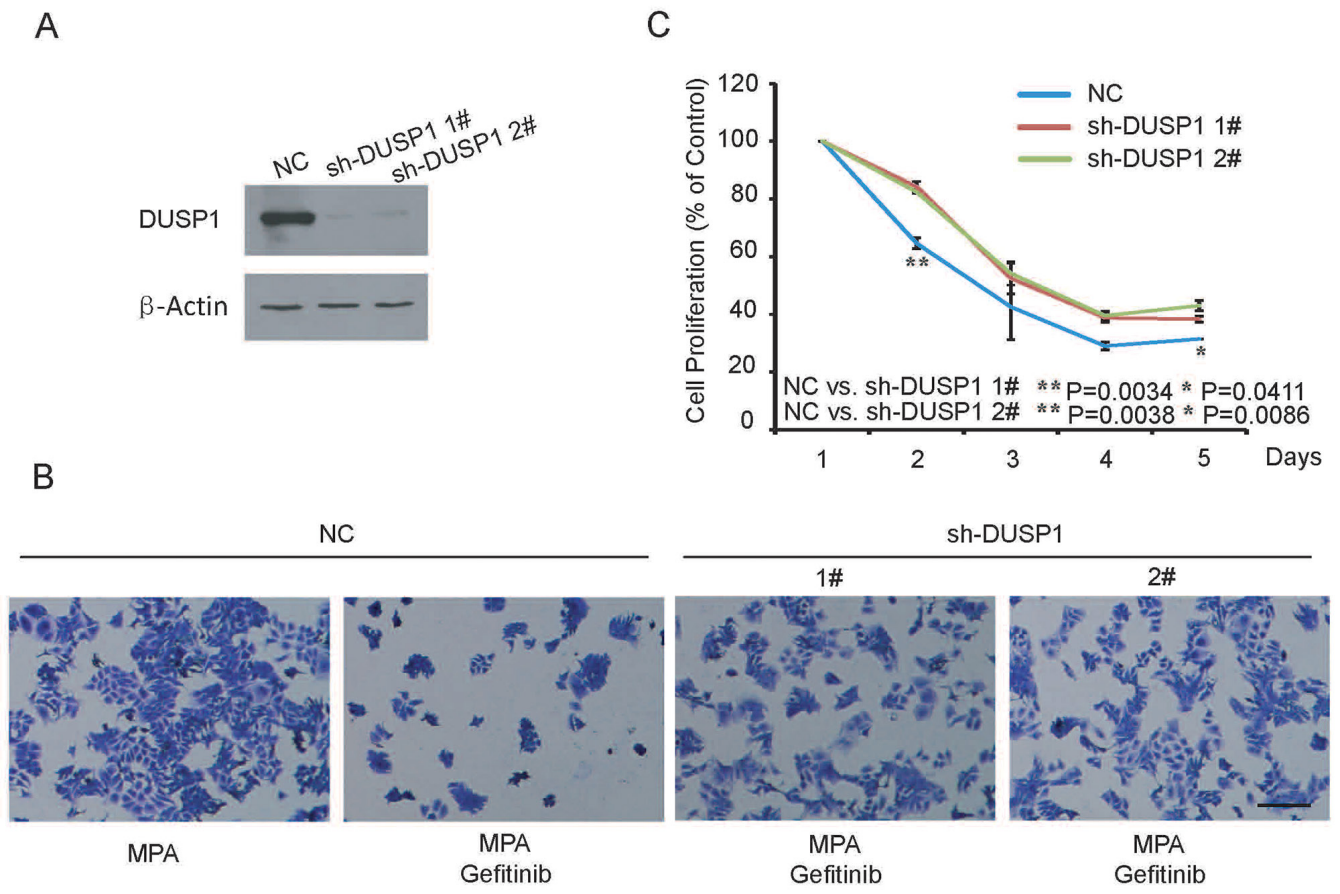
DUSP1 knockdown Ishikawa cells are resistant to therapeutic effects of combined Gefitinib and MPA treatment **(A)** Representative western blot shows DUSP1 levels in Ishikawa cells transfected with shRNAs 1# and 2# against DUSP1 and negative control shRNA. As shown, DUSP1 levels are significantly decreased in Ishikawa cells transfected by shRNAs 1# and 2# against DUSP1 than in Ishikawa cells transfected with negative control shRNA. **(B)** Representative image (x 200) shows viability of control and DUSP1 knockdown Ishikawa cells treated with 10μM MPA or 10μM Gefitinib plus 10μM MPA and stained with crystal violet on day 2. **(C)** Histogram plot shows relative percentage of control and DUSP1 knockdown Ishikawa cells treated with 10μM MPA, 10μM Gefitinib plus 10μM MPA or DMSO between 1-5 days. Data are representative of triplicate experiments.

In our previous study, we demonstrated that MPA treatment upregulated DUSP1, which subsequently decreased EC progression by dephosphorylating ERK1/2 [18]. Therefore, we investigated the role of DUSP1 in the combined Gefitinib and MPA treatment of EC cells. We established two stable DUSP1 knockdown Ishikawa cell lines by transfecting pLVX vector with two DUSP1 shRNAs, sh-DUSP1-1# and sh-DUSP1-2# (Figure 3A). CCK-8 assay showed that DUSP1 knockdown partly decreased the inhibition of growth and migration inhibition effects of Gefitinib and MPA combination from day 2 onwards (Figure 3B-3C). The partial response could be either due to partial down regulation of DUSP1 in knockdown cells or because DUSP1 pathway was part of multiple pathways involved in the anti-tumor effects of Gefitinib and MPA treatment. In spite of the partial effect, our study indicates that DUSP1 plays an important role in the anti-tumor effects of Gefitinib and MPA treatment combination.

### DUSP1 mediates effects of combined Gefitinib and MPA treatment in EC cells

**Figure 4:**
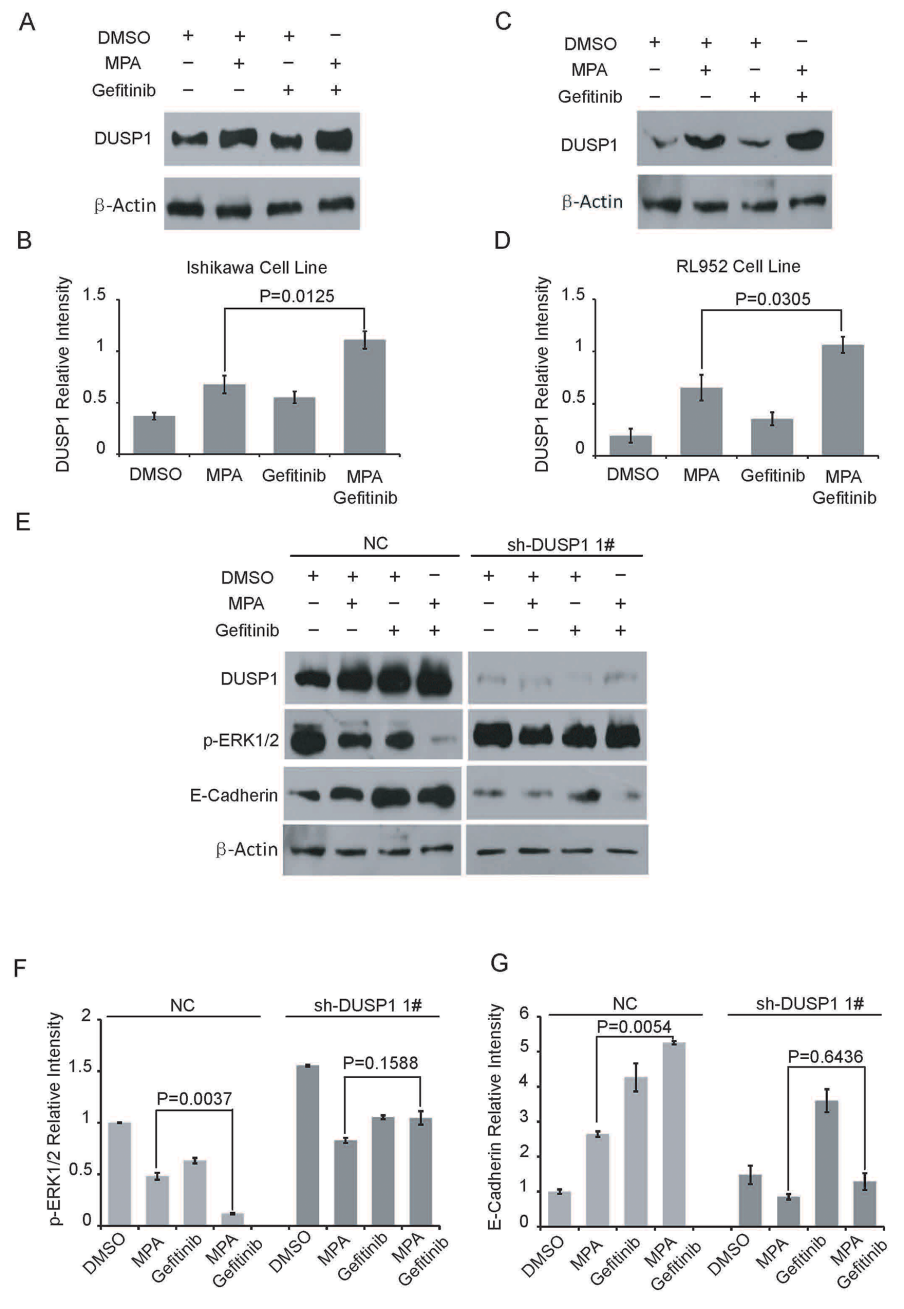
Gefitinib enhances MPA-mediated suppression of EC cells via DUSP1 **(A-D)** Representative western blots show DUSP1 (A, C) and β-Actin (B, D) levels in Ishikawa and RL952 cells treated with 5μM Gefitinib, 10μM Gefitinib, 10μM MPA, 10μM MPA plus 5μM Gefitinib or 10μM MPA plus 10μM Gefitinib for 24 h. **(E)** Representative western blots show DUSP1, p-ERK1/2, E-Cadherin and β-Actinlevels in parental and DUSP1 knockdown Ishikawa cells treated with 10 μM Gefitinib, 10μM MPA or 10μM MPA plus 10 μM Gefitinib for 24 h. **(F-G)** Histogram plots show p-ERK1/2 and E-Cadherin expression levels relative to β-Actin in parental and DUSP1 knockdown Ishikawa cells treated with 10 μM Gefitinib, 10μM MPA or 10μM MPA plus 10 μM Gefitinib for 24 h. Error bars represent the standard deviation (n=3).

We further investigated the role of DUSP1 in the anti-tumor effects of combined Gefitinib and MPA treatment. As previously shown [18], we observed DUSP1 induction upon MPA treatment of EC cells (Figure 4A-4D). But, Gefitinib treatment had no effect on DUSP1 protein levels after 24 h (Figure 4A-4D). However, DUSP1 protein levels increased in both Ishikawa and RL952 cells treated with a combination of Gefitinib and MPA than MPA alone (Figure 4B and 4D). These results indicated that Gefitinib further upregulated DUSP1 and therefore enhanced the anti-tumor effects of MPA in EC cells.

DUSP1 is activated by phosphorylation on two Serine residues, Ser359 and Ser364 by ERK1/2 in human embryonic kidney (HEK) 293 cells; on the other hand, DUSP1 inactivates ERK1/2 by dephosphorylation in human bladder cancer HT1197 cells [23–25]. In our previous study, shRNA-mediated knockdown of DUSP1 increased p-ERK1/2 levels, which correlated with increased proliferation of EC cells. Therefore, we investigated the status of p-ERK1/2 in the combined Gefitinib and MPA treatment of DUSP1 knockdown Ishikawa cell lines and their corresponding controls. As shown in Figure 4E and Figure 4F, Ishikawa cells treated with a combination of Gefitinib and MPA showed lower p-ERK1/2 levels than in MPA-treated parental Ishikawa cells (with DUSP1 expression). However, DUSP1 knockdown abolished the effects of the combined treatment and showed elevated phospho-ERK1/2 levels (Figure 4F).

We further demonstrated increased E-cadherin levels in Ishikawa cells treated with combination of Gefitinib and MPA than in EC cells treated with MPA alone (Figure 4E-4G). However, DUSP1 knockdown Ishikawa cells treated with a combination of Gefitinib and MPA showed low E-cadherin levels (Figure 4E, 4G). These results suggested that Gefitinib boosted E-cadherin upregulation by MPA through DUSP1, which correlated with inhibition of EC cell migration. These data suggest that DUSP1 mediates enhanced sensitivity of EC cells to combined treatment of Gefitinib and MPA.

**Figure 5:**
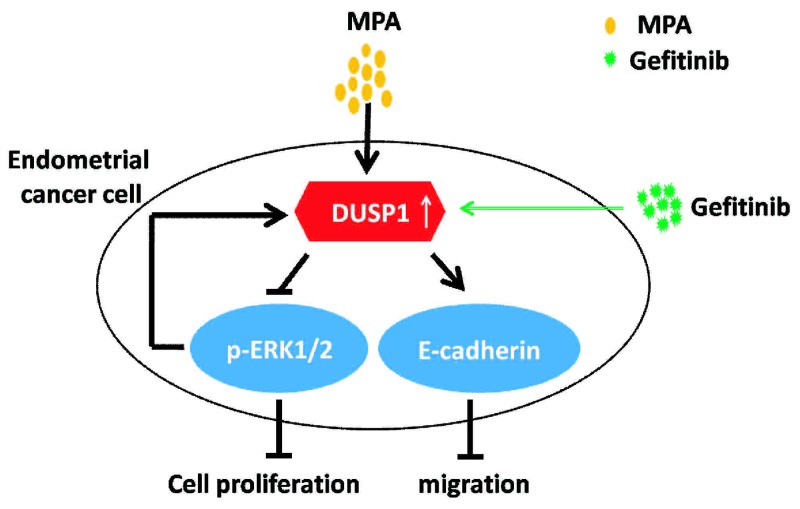
Schematic diagram illustrates mechanism of Gefitinib-mediated progestin therapy

As shown in Figure 5, Gefitinib enhances DUSP1 upregulation by MPA. In turn, DUSP1 inhibits phosphorylation of ERK1/2 and enhances E-cadherin expression. These changes result in decreased proliferation and migration of EC cells. Therefore, Gefitinib enhances the sensitivity of EC cells to MPA by enhancing DUSP1 upregulation.

## DISCUSSION

MPA and Gefitinib are effective drugs in breast and endometrial cancer therapy [12, 26]. Moreover, Gefitinib is an effective drug in therapy for prostate, gastric, colorectal and non-small cell lung cancers [27–30]. Our data demonstrates that Gefitinib enhances anti-tumor properties of MPA via DUSP1 in EC cells, both *in-vitro* and *in-vivo*. Gefitinib enhances the inhibition of p-ERK1/2 and upregulation of E-cadherin by MPA, resulting in decreased proliferation and migration, respectively. Combined Gefitinib and MPA treatment also inhibits *in vivo* growth of EC xenografts in nude mice. Moreover, the anti-tumor effects of combined Gefitinib and MPA treatment are mediated by DUSP1.

The mechanism of progestin therapy in EC involves (1) progesterone binding to the progesterone receptor (PR), which switches its conformation, dimerizes and translocates to the nucleus where it binds specific PR response elements in target genes and initiates gene expression in collaboration with various other transcription factors; and (2) progestins suppress MAPK and PI3K/AKT signaling pathways, there by inhibiting cell differentiation and growth [31, 32]. Gefitinib is an EGFR-TKI inhibitor, which inhibits Ras/Raf/MAPK, ERK1, ERK2 and PI3K/AKT signal transduction pathways that are involved in the regulation of cell growth, migration, adhesion, angiogenesis and apoptosis [33].

Hyperactivation of PI3K/AKT and MAPK/ERK pathways by phosphorylation are central to growth and progression of various solid tumors. Phosphorylated AKT dissociates from plasma membrane receptors and migrates to the cytoplasm and the nucleus, thereby regulating cell proliferation, differentiation and apoptosis by phosphorylating downstream targets such as GSK- 3, E2F, CDK, FKHR, Bad and caspase-9 [34, 35]. MAPK/ERK pathway regulates cancer cell growth, differentiation and survival by phosphorylating downstream substrates such as Elk-1, c-Myc, c-Fos, c-Jun, ATF, NF-κB and AP-1 [36–38]. E-Cadherin is a key indicator of the degree of epithelial to mesenchymal transition (EMT), which determines tumor invasion and metastasis [39].

DUSP1 is a member of the family of double specificity phosphatases, which phosphorylate tyrosine and threonine residues in MAP Kinases to inhibit cell growth, differentiation and apoptosis [35]. In pancreatic cancer, DUSP1 decreases tumor cell proliferation by inhibiting the MAPK/ERK pathway [36]. High expression of DUSP1 is an independent risk factor that determines prognosis of early lung cancer patients [40]. High expression of DUSP1 induces apoptosis in prostate cancer cells [38]. In our previous study, DUSP1 deficiency promotes EC progression via the MAPK/ERK pathway [18]. Tumor progression via EMT correlates with activities and interactions of WNT/TGFβ, Hedgehog, PI3K/AKT and MAPK signaling pathways [41]. In this study, we demonstrate that high DUSP1 expression correlates with EC cell migration and E-Cadherin expression. This suggests that DUSP1/E-Cadherin signaling axis regulates EMT.

In conclusion, our results show that Gefitinib augments progestin therapy sensitivity in EC cells by enhancing DUSP1 levels. Further pre-clinical and clinical trials are needed to validate the potential of combination of Gefitinib and MPA for EC treatment.

## MATERIALS AND METHODS

### Reagents and EC cell lines

Gefitinib (SML1657, Sigma, USA) and Medroxyprogesterone acetate (1378001, Sigma, USA) were dissolved in 100% Dimethylsulfoxide (DMSO) and used for *in vitro* or *in vivo* studies at concentrations not exceeding 0.1% DMSO.

Hec1A (Lot No. 58087755) and RL952 (Lot No. 62130010) human EC cell lines were purchased from ATCC (Manassas, VA, USA). The human EC cell line, Ishikawa, was obtained from our laboratory stock. Hec1A cells were grown in DMEM medium (SH30243.01B, Hyclone, USA) containing 10% fetal bovine serum (FBS; 16000044, Gibco, USA) and 100 mg/mL penicillin/streptomycin (CC004, M&C GENE, China) at 37°C and 5% CO_2_. RL952 and Ishikawa were cultured in DMEM/F12 (SH30023.01B, Hyclone, USA) containing 10 % FBS and 100 mg/mL penicillin/streptomycin at 37°C and 5% CO_2_. The medium was replenished every day.

### Generation of stable DUSP1 knockdown Ishikawa cell line

We used two shRNAs against DUSP1: (1#) 5´-CCACCATCTGCCTTGCTTACCTTAT-3´(sense); 5´-ATAAGGTAAGCAAGGCAGATGGTGG-3´(antisense); and (2#) 5´-AGTCCCAGGTGCTGGCTCCGCA CTG-3´ (sense); 5´-CAGTGCGGAGCCAGCACCTGGGACT-3´ (antisense). The negative control shRNA sequences were 5´-GTTCTTCCGAACGTGTCACGT-3´(sense) and 5´-ACGTGACACGTTCGGAAGAAC -3´ (antisense). All the shRNA sequences were synthesized and cloned into pGPU6/Neo by Gene Pharma, China. The shRNAs were transfected into 1×10^5^ Ishikawa cells with Lipofectamine 2000 (11668500, Invitrogen, USA), according to the manufacturer’s instructions. Stably transfected cells were selected in DMEM/F12 supplemented with 10% FBS and 700 μg/mL G418 (A1720, Sigma, USA) for 14 days. DUSP1 knockdown was confirmed by RT-PCR and western blotting.

### CCK-8 cell proliferation assay

We incubated 1000 EC cells (Ishikawa, RL952 and Hec1A) with different drug concentrations (0, 0.05, 0.5, 5, 10 and 20μM Gefitinib; 0, 0.01, 0.1, 1, 10 and 20μM MPA) in 96-well plates for 4 consecutive days. Then, we added 10 μL Cell Counting Kit-8 solution (CCK-8; CK04, Dojindo, Japan) to each well and measured the color intensity in a microplate reader (Tecan Infinite 200) at 490 nm. Experiments were performed in triplicate.

### Transwell invasionassay

In the Transwell invasion assay, 1×10^5^ EC cells were seeded in the upper chamber of Costar Transwell culture plates (24-well plates, 8 μm) in 100 μL DMEM/F12 without FBS, whereas 600 μL of DMEM/F12 supplemented with 10 % FBS was added to the lower chamber. After incubation for 24 h, cells in the upper membrane that failed to migrate were wiped with a cotton swab. Then, the cells in the lower chamber were fixed with 4% paraformaldehyde, stained with 0.5% crystal violet and counted under a light microscope in five high power fields (200×). All experiments were performed in triplicate.

### Western blotting

Total protein lysates were prepared from EC cells and quantified by the Bradford method (5000201, BIO-RAD, USA). Equal amounts of cell lysates (20 μg) were separated by 12% SDS-PAGE and transferred onto nitrocellulose membranes (Merck Millipore Ltd) (1.5 h-2 h for run; 2 h for transfer). Then, the blots were blocked with 5% nonfat milk and incubated with primary antibodies against DUSP1 (1:1000, sc-1102, Santa, USA), p-ERK1/2 (1:1000, #9102, Cell Signaling, USA), E-Cadherin (1:1000, #3195, Cell Signaling, USA), β-Actin (1:2000, #4970, Cell Signaling, USA) at 4°C overnight. Then, the blots were incubated with HRP-conjugated goat anti-rabbit secondary antibody (ZB2301, ZSGB-BIO, Beijing) for 2 h at room temperature and then developed with ECL (P1020, Applygen, China). The band intensities were determined with the Bio-Rad imaging system (Hercules, CA, USA).

### *In vivo* xenograft tumorigenicity assay

Six-week-old BALB/c female athymic nude mice (SPF Laboratory Animal Technology Co. LTD., Beijing, China) were subcutaneously injected in the right flank with 2 × 10^6^ Ishikawa cells in 0.1 ml PBS. After seven days, the mice were randomly divided into four groups (*n* = 6) and intraperitoneally injected with(i) 100μl DMSO with 200 μL saline; (ii) 500 mg/kg MPA in 300 μL saline; (iii) 250 mg/kg Gefitinib in 300 μL saline; and (iv) combination (both 500 mg/kg MPA and 250 mg/kg Gefitinib in 300 μL saline), every three days. After 3 weeks, the mice were euthanized and tumor volumes were measured with calipers [tumor volume=(length × width^2^)/2]. All animal studies were conducted according to protocols approved by the Animal Ethics Committee of Peking University People’s Hospital.

### Statistical analysis

Statistical analyses were conducted by SPSS 19.0 (SPSS Inc., Chicago, IL, USA) and GraphPad Prism 5 (SanDiego, CA, USA) softwares. Data are represented as mean ±S.D. Statistical significance was determined by one-way ANOVA with a Bonferroni post hoc test except in Figure 4F and 4G, where the data was measured by two-way ANOVA with a Bonferroni post hoc test. P< 0.05 was considered statistically significant.
